# Tri-modal PSMA PET/CT-MRI-US fusion biopsy for clinically significant prostate cancer: a prospective paired diagnostic study

**DOI:** 10.1007/s00259-026-08181-x

**Published:** 2026-09-15

**Authors:** Chen Huang, Lichen Jin, Yizhang Sun, Menglin Wu, Xuefeng Zhang, Xiaofeng Xu, Xuefeng He, Feng Qiu, Ximing Wang, Lingchuan Guo, Renpeng Huang, Jihui Li, Shengming Deng, Yuxin Lin, Xuedong Wei, Bin Zhang, Yuhua Huang

**Affiliations:** 1 Department of Urology, The First Affiliated Hospital of Soochow University, 899 Pinghai Road, Suzhou, 215006 China; 2 School of Computer Science and Technology, Nanjing Tech University, Nanjing, 211816 China; 3 Carbon Medical Device Ltd, Shenzhen, China; 4 Department of Radiology, The First Affiliated Hospital of Soochow University, Suzhou, 215006 China; 5 Department of Pathology, The First Affiliated Hospital of Soochow University, Suzhou, 215006 China; 6 Department of Nuclear Medicine, The First Affiliated Hospital of Soochow University, 899 Pinghai Road, Suzhou, 215006 China

**Keywords:** Clinically significant prostate cancer, PSMA PET/CT, Prostate biopsy, Risk score, Fusion biopsy, Multimodal imaging

## Abstract

**Purpose:**

Multiparametric MRI (mpMRI)-ultrasound (US) fusion biopsy misses 8%-20% of clinically significant prostate cancer (csPCa), leaving a detection gap that prostate-specific membrane antigen (PSMA) PET/CT may help close. Whether incorporating PSMA into an MRI-US fusion platform improves csPCa detection has not been evaluated in a prospective paired design. We compared tri-modal PSMA PET/CT-MRI-US fusion biopsy with MRI-US fusion biopsy and systematic biopsy.

**Methods:**

In this prospective, single-center, and paired diagnostic accuracy study (STARD 2015; ChiCTR2400089436), 308 biopsy-naive men underwent tri-modal fusion biopsy, MRI-US fusion biopsy, and systematic biopsy in a single session. The primary endpoint was the absolute difference in patient-level csPCa detection (International Society of Urological Pathology grade group ≥ 2) between tri-modal and MRI-US fusion biopsies.

**Results:**

Tri-modal fusion detected 4.9% more csPCa than MRI-US fusion biopsy (95% confidence interval 2.4%-7.3%; *P* < 0.001; number needed to biopsy 20.5). The most significant gains were observed for PSMA-positive lesions < 10 mm (8.3%) and Prostate Imaging Reporting and Data System (PI-RADS) 4 lesions (5.8%), and this benefit persisted in multivariate generalized estimating equation models. The PSMA imaging-based risk score (area under the curve, 0.933) showed higher discrimination than the PRIMARY and molecular imaging PSMA scores. In a retrospective simulation using the test set, a risk-guided approach would have avoided approximately 42% of biopsies while missing 2.3% of csPCa. Limitations include the single-center design and the small test set.

**Conclusion:**

Tri-modal fusion was superior to MRI-US fusion biopsy in detecting csPCa, particularly in small PSMA-positive and PI-RADS 4 lesions. A PSMA-derived risk score enabled effective stratification, and in the test set, a simulated risk-guided approach showed potential for biopsy reduction. These findings require multicenter validation before clinical adoption.

**Trial registration:**

ChiCTR2400089436 (Chinese Clinical Trial Registry, http://www.chictr.org.cn/, registered 2024-09-09).

**Supplementary Information:**

The online version contains supplementary material available at https://doi.org/10.1007/s00259-026-08181-x.

## Introduction

Maximizing the detection of clinically significant prostate cancer (csPCa) while avoiding unnecessary biopsies remains a central diagnostic challenge. Multiparametric magnetic resonance imaging (mpMRI) combined with fusion biopsy has improved this balance allowing approximately 30% of patients with negative mpMRI findings to safely defer biopsy [1, 2]. However, mpMRI fails to detect 8%-20% of csPCa, which are identified only via systematic biopsy (SB) [3–5]. This diagnostic gap is particularly relevant for Prostate Imaging Reporting and Data System (PI-RADS) 3 and 4 lesions, for which diagnostic uncertainty remains greatest.

Prostate-specific membrane antigen (PSMA) PET/CT exploits receptor overexpression in aggressive disease to reveal MRI-occult lesions [6, 7]. The two modalities detect different biological features. mpMRI detects structural disruption, whereas PSMA PET targets receptor expression that is associated with tumor aggressiveness [8–10]. Published discordance rates between PSMA PET and mpMRI range from 30% to 50% [8, 9], reflecting genuinely complementary biological characteristics rather than a diagnostic failure. PSMA-only lesions carry csPCa rates of 26%-39%, and MRI-only lesions have those of 15%–20% [7, 10]. The PRIMARY trial [8] and a subsequent meta-analysis [7] demonstrated the additive diagnostic value of PSMA PET over mpMRI alone, but no prior study has evaluated whether incorporating PSMA into a real-time MRI-US fusion biopsy platform further improves csPCa detection in a prospective paired design.

Translating imaging findings into accurate biopsy results requires precise spatial correspondence between a preoperative image and a real-time targeting plane. However, biopsy outcomes frequently diverge from imaging predictions. A lesion scored as highly suspicious may yield benign tissue if the needle misses the tumor core, and csPCa can be found in systematic cores from regions deemed negative on imaging. This discordance partly reflects the inherent difficulty of needle placement. Conventional MRI-US fusion relies on visual or software-assisted registration of preoperative MRI to intraoperative transrectal ultrasound (TRUS), but this process is susceptible to prostate deformation, patient movement, and inter-operator variability in probe positioning [11]. MRI and TRUS images are acquired in different planes and under different conditions (supine vs. lithotomy), and the resulting registration errors can exceed the diameter of small clinically significant tumors, potentially leading to missing samples.

We designed a prospective paired diagnostic study with two complementary objectives. The first was to compare tri-modal fusion biopsy head-to-head with standard MRI-US fusion biopsy and SB for csPCa detection in a single biopsy session. This paired design directly quantifies the incremental diagnostic yield contributed by PSMA-informed targeting. The second objective was to develop and internally validate a PSMA imaging-based risk score combining quantitative PET parameters with clinical and MRI variables and to identify patients in whom biopsy could be safely deferred. These two objectives are closely linked. The diagnostic accuracy comparison provides the evidence base for tri-modal fusion, whereas the risk score, combined with PI-RADS findings and prostate-specific antigen density (PSAD), translates imaging findings into a clinical decision-making framework.

## Materials and methods

### Study design and participants

**Fig. 1:**
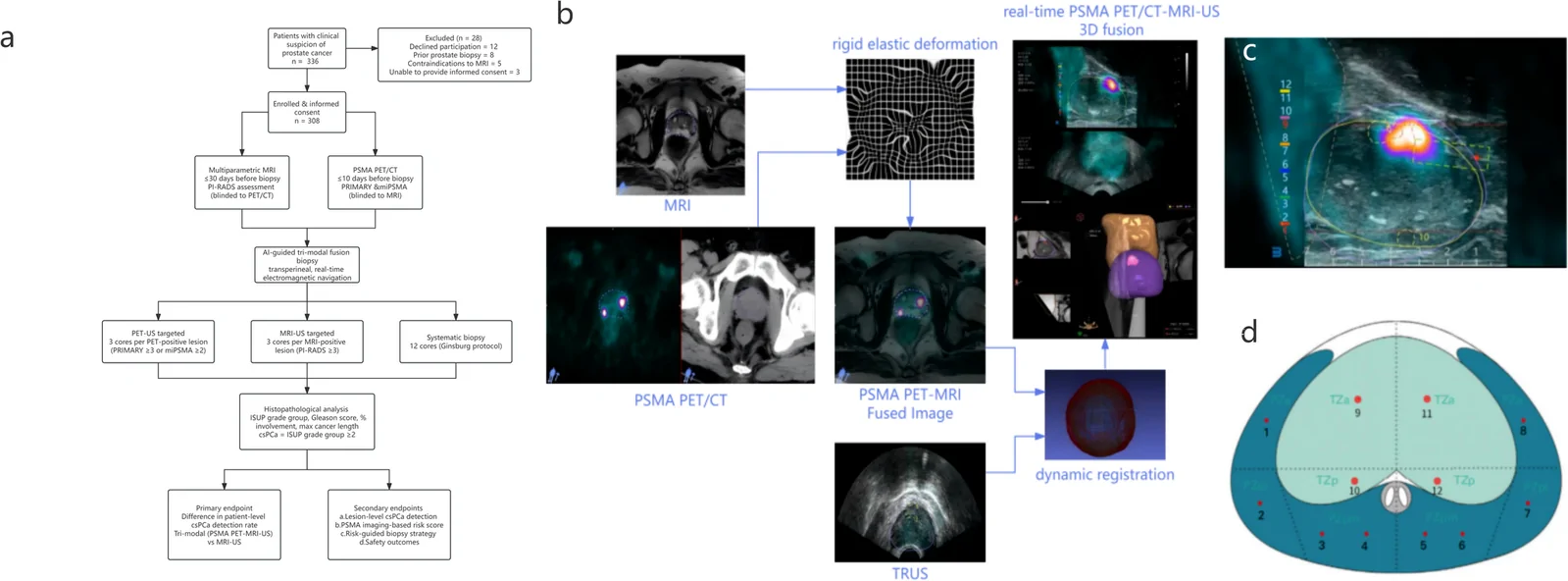
Study flow diagram and tri-modal fusion workflow. (**a**) Flow diagram showing patient enrollment, imaging, biopsy procedures, and analysis flow. All image readers and pathologists were blinded to other modalities. (**b**) Tri-modal fusion workflow including automatic prostate segmentation, iterative closest point registration, and electromagnetic tracking. (**c**) Sagittal view of the fused ultrasound image, displaying the prostate contour, the suggested needle insertion point (red dot) and a lesion with intense tracer uptake. (**d**) Biopsy core distribution diagram based on the Ginsburg protocol

This prospective, single-center diagnostic accuracy study used a paired design to compare three biopsy approaches for csPCa detection in biopsy-naive men: tri-modal PSMA PET/CT-MRI-US fusion biopsy, MRI-US fusion biopsy, and SB (Fig. 1a). Eligible patients had suspected prostate cancer based on suspicious findings on digital rectal examination, imaging findings, or elevated prostate-specific antigen (PSA) levels. Exclusion criteria included a prior diagnosis of prostate cancer, prior prostate biopsy, inability to provide informed consent, and contraindications to MRI. Of the 336 patients assessed, 308 were enrolled between October 2024 and March 2026, whereas 28 were excluded (Fig. 1a). The study followed the “Standards for Reporting of Diagnostic Accuracy Studies” (STARD) 2015 guidelines (ChiCTR2400089436). The interval from imaging to biopsy was ≤ 30 days for mpMRI and ≤ 10 days for PSMA PET/CT. Detailed inclusion and exclusion criteria are provided in Supplementary Table S1 and the schedule of events is presented in Supplementary Table S2.

The Institutional Review Board of the First Affiliated Hospital of Soochow University approved this study (No. 2024173). We registered the protocol with the Chinese Clinical Trial Registry (ChiCTR2400089436; registered 2024-09-09), and all participants provided written informed consent before enrollment.

### Sample size calculation

We calculated the sample size for a paired superiority design using McNemar’s test. Based on the PRIMARY trial [8], we assumed a discordant-pair rate of 25% and a minimal clinically important difference (MCID) of 10% for the absolute detection advantage, with a two-sided α of 0.05 and 90% power [12]. These parameters yielded a minimum required sample size of 246 participants; Allowing for a 25% rate, the target sample size was increased to 308.

### Imaging protocols

mpMRI was performed within 30 days before biopsy using a 3.0 T scanner (MAGNETOM Skyra; Siemens). The protocol comprised T1-weighted, T2-weighted, and diffusion-weighted sequences (b = 0–1600 s/mm2) and dynamic contrast-enhanced sequences. Two radiologists blinded to the PET data scored lesions according to PI-RADS v2.1. PSMA PET/CT (18 F-PSMA-1007, 1.85 MBq/kg, uptake time 90 ± 10 min) was performed within 10 days before biopsy using a Biograph Vision 600 scanner. Nuclear medicine specialists blinded to the MRI data, assigned PRIMARY scores (positive ≥ 3) and molecular imaging PSMA (miPSMA) scores (positive ≥ 2) [13]. The maximum standardized uptake value (SUVmax) ratio was defined as the lesion SUVmax divided by the parotid gland SUVmax, consistent with the definition of miPSMA grade 3 (uptake exceeding that of the parotid gland) [13].

### AI-guided tri-modal fusion workflow

The tri-modal fusion workflow (patent CN119257741B) [14] proceeds in three stages. A convolutional neural network (CNN) first segments the prostate on preoperative CT and MRI. The resulting surfaces are converted into point clouds and rigidly aligned using an iterative closest point (ICP) algorithm. The optimal rigid transformation is determined by minimizing:

$$T_{\theta^\ast}=arg\underset\theta{min}\left(-\lambda\bullet L_{MI}\left(T_\theta\left(I_{CT}\right),I_{MRI}\right)-L_{Dice}\left(T_\theta\left(S_{CT}\right),S_{MRI}\right)\right)$$

where $\:{\mathrm{L}}_{\mathrm{M}\mathrm{I}}$ is the mutual information loss, $\:{\mathrm{L}}_{\mathrm{D}\mathrm{i}\mathrm{c}\mathrm{e}}$ is the Dice overlap loss and λ is the weighting factor balancing the two terms. Because PSMA PET and CT share the same coordinate system, the PSMA PET image is then mapped into MRI space. During biopsy, the same CNN segments real-time TRUS images, and point-cloud registration under electromagnetic tracking is performed by solving the following equation:

$$\begin{array}{c}T_{R^\ast,t^*}=arg\;min\sum_{R,t\;i=1}^n||R\cdot\rho_i+t-q_j||^2\cdot\end{array}$$

where R and t denote the rotation matrix and translation vector, respectively; pi (i = 1, …, n) denotes the i-th point of the TRUS prostate contour point set in electromagnetic space; qj denotes its corresponding point in the MRI prostate contour point set; and n is the total number of TRUS contour points. Segmentation and registration are automated, although the operator may manually refine the segmentation boundaries when the automated output is visually suboptimal. This procedure achieves real-time PSMA PET/CT-MRI-US tri-modal fusion navigation [11] and ensures precise needle targeting (Fig. 1b). The Carbon AI fusion platform integrates deep learning-based segmentation, point-cloud registration, and electromagnetic tracking to effectively combine PSMA PET/CT, MRI, and real-time ultrasound data.

### Biopsy and histopathology

For tri-modal fusion biopsy (Fig. 1c), three cores were obtained from each PET-positive lesion and three from each MRI-positive lesion. Co-localized lesions (positive on both modalities) therefore received six cores. For MRI-US fusion biopsy, three cores were obtained from each MRI-positive lesion. whereas for SB, 12-core systematic sampling was performed (Fig. 1d). All patients underwent all three biopsy approaches in a single session. All procedures were performed using a transperineal approach under real-time fusion guidance. csPCa was defined as Grade Group ≥ 2 [15]. Pathologists blinded to the imaging findings and biopsy approach classified all biopsy cores.

### Outcome measures

The primary endpoint was the difference in patient-level csPCa detection rates between tri-modal and MRI-US fusion biopsies. Secondary endpoints included lesion-level detection rates, the diagnostic performance of the PSMA-derived risk score, and a simulated risk-guided biopsy strategy combining the PSMA score, PI-RADS, and PSAD.

### Statistical analysis

We used a two-sided exact McNemar’s test for the primary comparison. Superiority was declared when the lower bound of the 95% confidence interval (CI) for the absolute difference exceeded 0. Non-inferiority of combined fusion versus SB was assessed using a one-sided 97.5% CI (non-inferiority margin, = 5%) [16]. At the lesion level, generalized estimating equations (GEE) with a logit link, an exchangeable correlation structure, and robust standard errors were used to account for within-patient clustering (536 lesions from 308 patients; estimated working correlation, 0.12). Prespecified subgroup interaction tests were Bonferroni-corrected (α = 0.0083), whereas area under the curve (AUC) comparisons (using DeLong’s test) were considered exploratory.

For development of the PSMA score, the cohort was divided into training (70%, *n* = 216) and test (30%, *n* = 92) sets using stratified random sampling. Predictors were selected via stepwise logistic regression (entry *P* < 0.10, retention *P* < 0.05) from least absolute shrinkage and selection operator candidates (1-standard-error rule; the resulting nomogram is shown in Supplementary Fig. S1) and validated with 2000 bootstrap iterations. The bootstrap procedure was used to estimate CIs and calculate the optimism-corrected AUC, indicating minimal overfitting [13]. Discrimination was assessed using the area under the receiver operating characteristic (ROC) curve and calibration was evaluated with the Hosmer-Lemeshow test; 10-fold cross-validation provided an additional internal validation. Continuous variables were assessed for normality using the Shapiro-Wilk test, non-normally distributed data are presented as the median interquartile range (IQR) and were compared using the Mann-Whitney U test. No primary analysis variables had missing values. Missing secondary variables (< 2%) were multiply imputed using multiple imputation by chained equations (m = 5). All analyses were performed using R version 4.2.3.

### Bias control

Blinding was maintained across specialties. Nuclear medicine physicians had no access to MRI data, radiologists had no access to PET data, and pathologists were blinded to all imaging and biopsy information. PRIMARY and miPSMA scores were assigned without reference to MRI findings. Urologists received only the lesion coordinates for biopsy guidance. This was a within-patient paired design, wherein all 308 patients underwent all three biopsy strategies (tri-modal fusion biopsy, MRI-US fusion biopsy, and SB) during a single session, enabling direct within-patient comparisons rather than between-group randomization.

## Results

### Study population and baseline characteristics

Of the 308 patients, 124 (40.3%) had csPCa. Compared with non-csPCa patients, those with csPCa were older (median 70 vs. 69 years, *P* = 0.007), had higher PSA levels (10.82 vs. 9.19 ng/mL, *P* = 0.007), and PSAD (0.31 vs. 0.16 ng/mL2, *P* < 0.001), smaller prostate volume (32.6 vs. 54.6 mL, *P* < 0.001), and higher SUVmax ratio (0.91 vs. 0.41, *P* < 0.001; Table 1). PI-RADS, PRIMARY, and miPSMA scores were also significantly higher in the csPCa group (all *P* < 0.001). Concordant lesions were more prevalent in the csPCa group (66.9% vs. 15.2%), whereas double-negative and PET-/MRI+ lesion phenotypes predominated in the non-csPCa group.

**Table 1:** Baseline characteristics of the study population according to csPCa status

Variable	csPCa negative	csPCa positive	*P*-value	SMD
n	184	124		
Age, years (median [IQR])	69.00 [62.00, 73.00]	70.00 [65.00, 75.00]	0.007	0.327
PSA, ng/ml (median [IQR])	9.19 [6.30, 13.28]	10.82 [6.59, 19.67]	0.007	0.494
PSAD, ng/ml² (median [IQR])	0.16 [0.10, 0.24]	0.31 [0.20, 0.62]	< 0.001	0.836
PV, ml (median [IQR])	54.57 [39.54, 74.34]	32.58 [25.95, 43.34]	< 0.001	0.831
SUVmax (median [IQR])	8.27 [6.57, 10.69]	16.75 [11.03, 30.92]	< 0.001	1.044
SUVmax ratio (median [IQR])	0.40 [0.30, 0.53]	0.91 [0.51, 1.48]	< 0.001	1.050
f/tPSA (median [IQR])	0.16 [0.13, 0.22]	0.12 [0.09, 0.15]	< 0.001	0.645
PI-RADS, n(%)			< 0.001	1.301
1–2	39 (21.2)	2 (1.6)		
3	89 (48.4)	22 (17.7)		
4	52 (28.3)	68 (54.8)		
5	4 (2.2)	32 (25.8)		
PRIMARY, n(%)			< 0.001	1.711
1	26 (14.1)	0 (0.0)		
2	73 (39.7)	6 (4.8)		
3	31 (16.8)	5 (4.0)		
4	25 (13.6)	28 (22.6)		
5	29 (15.8)	85 (68.5)		
miPSMA, n(%)			< 0.001	1.407
0–1	130 (70.7)	31 (25.0)		
2	52 (28.3)	34 (27.4)		
3	2 (1.1)	59 (47.6)		
Imaging phenotype, n(%)			< 0.001	1.844
Concordant				
Fully concordant	28 (15.2)	83 (66.9)		
Partially concordant	15 (8.2)	14 (11.3)		
Double negative	26 (14.1)	0 (0.0)		
Discordant				
Different location	29 (15.8)	21 (16.9)		
PET-/MRI+	72 (39.1)	4 (3.2)		
PET+/MRI-	14 (7.6)	2 (1.6)		

Data are presented as median [IQR] or n (%). *SMD* standardized mean difference, *PSAD* prostate-specific antigen density, *SUVmax* maximum standardized uptake value, *SUVmax* *ratio* SUVmax of the lesion divided by SUVmax of the parotid gland, *PI-RADS* Prostate Imaging Reporting and Data System, *miPSMA *molecular imaging PSMA score, *IQR* interquartile range, *PV* prostate volume, *f/tPSA* free/total PSA ratio

The 28 excluded patients comprised 12 who declined participation, 8 had had prior prostate biopsy, 5 had contraindications to MRI (e.g., pacemaker, claustrophobia), and 3 were unable to provide informed consent (Fig. 1a). The median age of enrolled patients was 69 years (IQR 65–73), the median PSA level was 9.68 ng/mL (IQR 7.32–13.41), and the median prostate volume was 44.0 mL (IQR 30.5–65.2). PI-RADS 4 was the most common index lesion category (39.0%), followed by PI-RADS 3 (36.0%) and PI-RADS 5 (11.7%).

### Primary endpoint and subgroup analyses

**Fig. 2:**
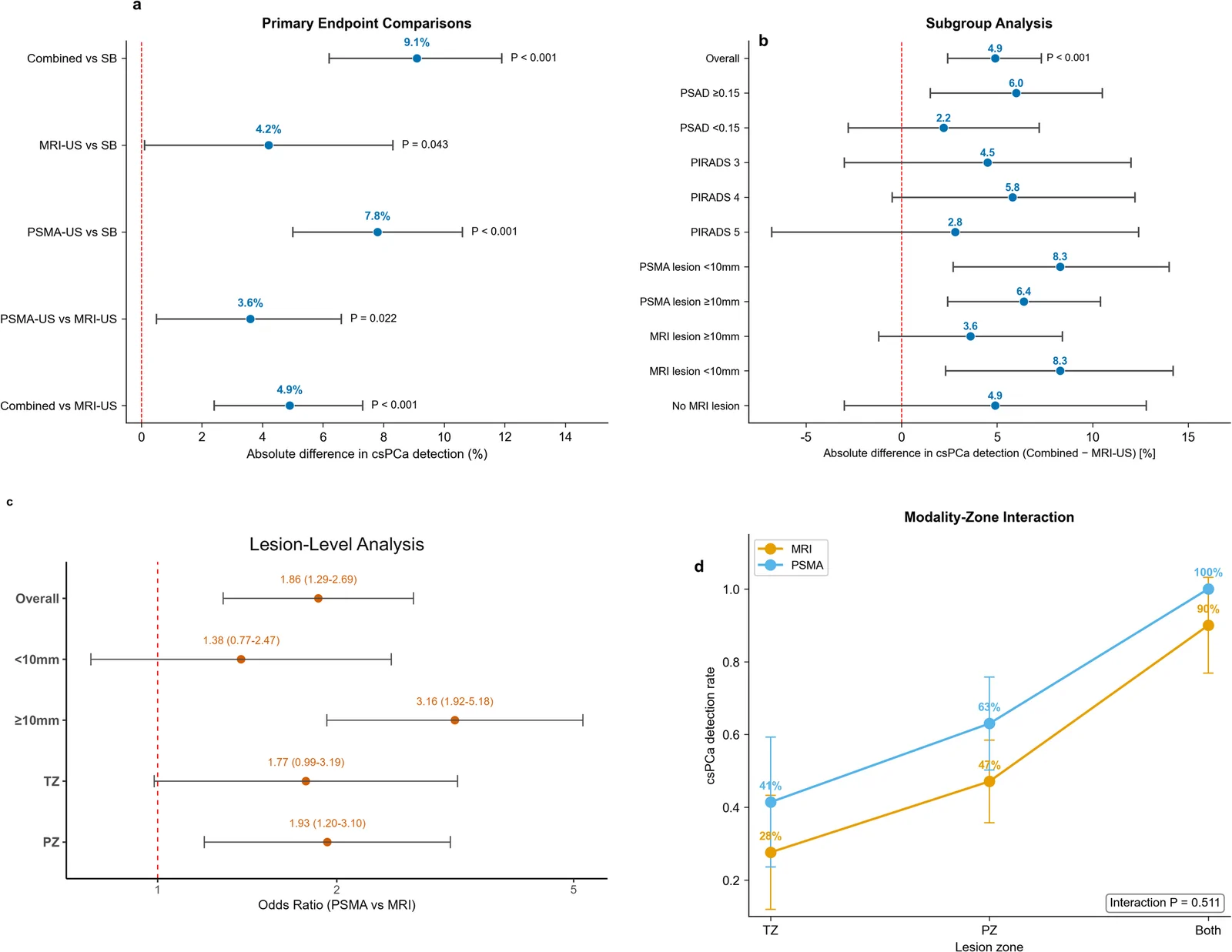
Primary endpoint and subgroup analyses. (**a**) Forest plot of absolute differences in csPCa detection rates between biopsy strategies (combined vs. MRI-US, PSMA-US vs. MRI-US, PSMA-US vs. SB, MRI-US vs. SB, and combined vs. SB). Points represent absolute percentage differences; error bars indicate 95% confidence intervals (CIs). P values are shown on the right. (**b**) Forest plot of subgroup analyses showing the absolute difference in csPCa detection between the combined strategy and MRI-US fusion biopsy. Subgroups include PSAD, PI-RADS category, PSMA lesion size, and MRI lesion size. (**c**) Lesion-level forest plot of ORs (PSMA vs. MRI) for csPCa detection from unadjusted GEE models. Estimates are shown for all lesions, by lesion size (< 10 mm vs. ≥10 mm), and lesion location (transition zone [TZ] vs. peripheral zone [PZ]). (**d**) Interaction plot of csPCa detection rates according to lesion location (TZ, PZ, and Both) and imaging modality (MRI vs. PSMA). The P-value for the modality-by-zone interaction from the GEE model is shown

In the paired analysis, tri-modal fusion biopsy detected csPCa in 119 patients versus 104 detected by MRI-US fusion biopsy. Fifteen patients were identified by tri-modal fusion biopsy but not by MRI-US fusion biopsy, whereas no patient was identified by MRI-US fusion biopsy alone (McNemar *P* < 0.001; Fig. 2a). The number needed to biopsy (NNB) was 20.5, indicating that approximately 20 patients would need to undergo tri-modal fusion biopsy to detect one additional csPCa beyond what MRI-US fusion biopsy alone would identify.

No csPCa cases were detected exclusively by MRI-US fusion biopsy alone because the tri-modal approach sampled all MRI-visible lesions in addition to PSMA-positive/MRI-negative lesions. Of the 124 patients with csPCa, 5 were detected exclusively by SB and missed by both fusion approaches, indicating that these tumors were not visible on either PSMA PET or MRI.

Prespecified subgroup analyses (Fig. 2b) showed the greatest absolute increases in csPCa detection for PSMA-positive lesions < 10 mm (8.3%, 95% CI 2.6%-14.1%, *P* = 0.013), PI-RADS 4 lesions (5.8%, 1.5%-10.1%, *P* = 0.023), and patients with PSAD ≥ 0.15 (6.0%, 2.8%-9.3%, *P* = 0.001). Differences were not significant for PI-RADS 3 lesions, PI-RADS 5 lesions, patients with PSAD < 0.15, or patients without PSMA-positive lesions (Supplementary Table S3). Among patients without MRI-visible lesions, the combined strategy showed a nonsignificant trend toward improved csPCa detection (absolute difference 4.9%; 95% CI -1.9% to 11.6%).

### Lesion-level analysis

Among non-co-localized lesions, the csPCa rate was 39.3% for PSMA-only vs. 17.5% for MRI-only (Supplementary Table S4). GEE analysis (Fig. 2c) showed that PSMA-positive lesions had higher odds of csPCa than MRI-positive lesions (unadjusted odds ratio [OR] = 1.86, 95% CI 1.29–2.69, *P* < 0.001). After adjusting for PSAD and age, the OR was 1.86 (1.24–2.80); further adjustment for PI-RADS yielded an OR of 1.97 (1.27–3.08). The advantage was more pronounced for lesions > = 10 mm (OR = 3.16, 1.92–5.18) but also evident for those < 10 mm (OR = 1.38, 0.77–2.47). The modality-zone interaction was not significant (*P* = 0.511; Fig. 2d).

Among the 390 unique targeted lesions, 146 (37.4%) were co-localized (positive on both PSMA PET/CT and mpMRI), 107 (27.4%) were PSMA-only, and 137 (35.1%) were MRI-only. Tri-modal fusion biopsy enabled sampling of all 390 lesions (1170 cores), whereas MRI-US fusion biopsy allowed sampling of the 283 MRI-positive lesions (849 cores). The additional 321 cores from PSMA-only lesions represented the median of three extra cores per patient (IQR, 3–3; 94 patients had PSMA-only lesions). Among non-co-localized lesions, the csPCa detection rate was significantly higher for PSMA-only than for MRI-only lesions (39.3% vs. 17.5%; OR = 3.04, 95% CI 1.69–5.47; *P* = 0.0002, Fisher’s exact test) (Supplementary Fig. S2). Among all PSMA-positive lesions, the SUVmax ratio predicted csPCa with an AUC of 0.735 (95% CI 0.673–0.797; Supplementary Fig. S3), with a higher SUVmax ratio associated with csPCa (median 0.85 vs. 0.50; *P* < 0.001, Supplementary Fig. S4). Co-localized lesions had the highest csPCa detection rate (62.3%), which was significantly higher than that of PSMA-only lesions (OR = 2.56, 95% CI 1.53–4.27; *P* = 0.0003).

Within PSMA-only lesions, csPCa was detected in 33.3% (24/72) of lesions < 10 mm and 51.4% (18/35) of lesions ≥ 10 mm. By prostate zone, csPCa detection rates were 36.5% (19/52) in the transition zone and 41.8% (23/55) in the peripheral zone (Supplementary Fig. S5).

### PSMA score and risk-guided strategy

**Fig. 3:**
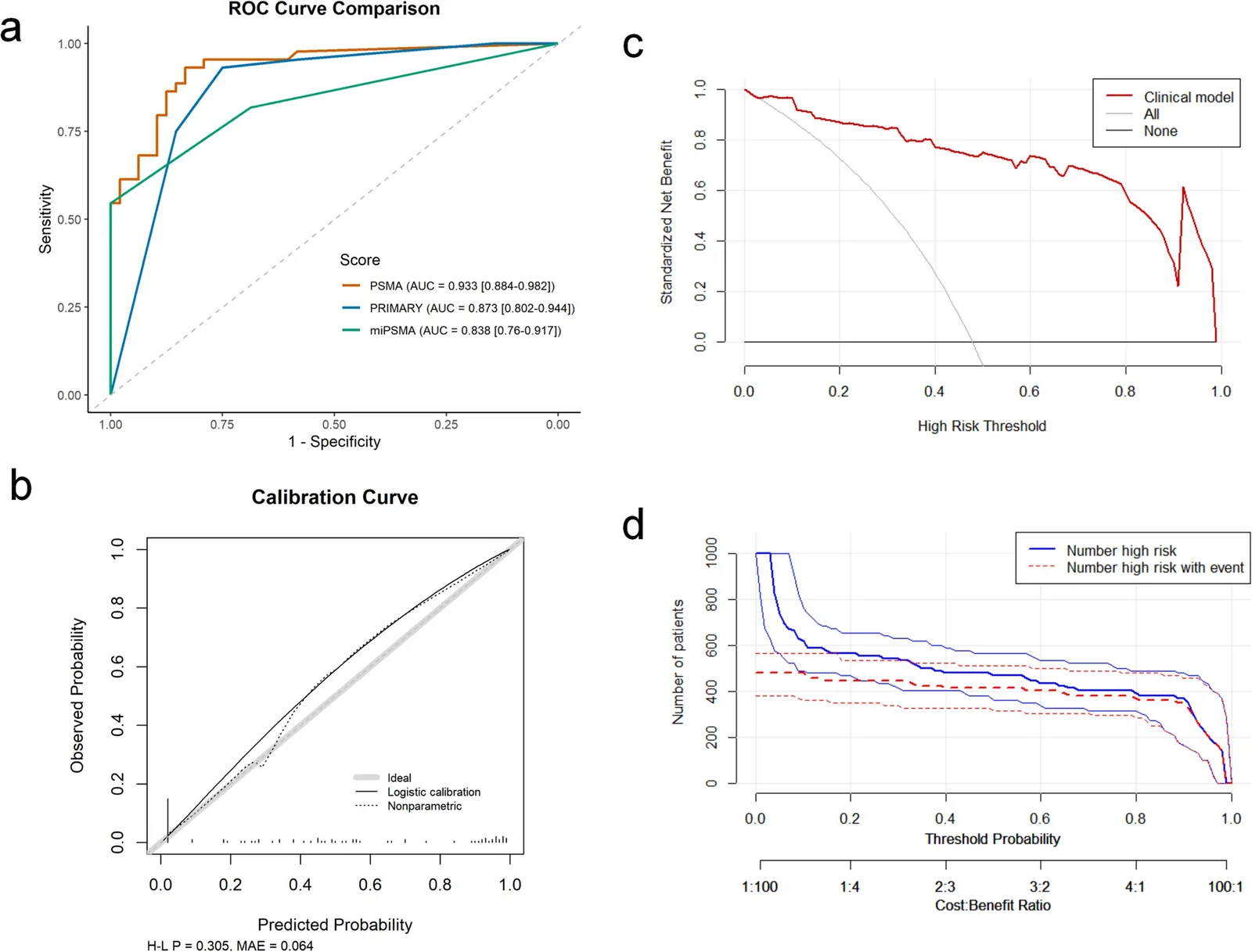
Performance of the PSMA-based risk score and clinical model. (**a**) ROC curves comparing the PSMA, PRIMARY, and miPSMA scores for csPCa detection in the test set. AUC values with 95% CIs are shown in the legend. DeLong test P-values were as follows: PSMA vs. PRIMARY = 0.030; PSMA vs. miPSMA = 0.006. (**b**) Calibration curve of the PSMA score in the test set. The fitted calibration curve and the ideal 45° reference line are shown. The Hosmer–Lemeshow P value and mean absolute error (MAE) are displayed. (**c**) Decision curve analysis comparing the risk-based biopsy strategy versus default strategies (“biopsy all” and “biopsy none”). Standardized net benefit is plotted against the threshold probability for high-risk threshold classification. Shaded areas represent bootstrap CIs. (**d**) Clinical impact curves for the clinical model. The numbers of patients classified as high risk (blue line) and the number of those with csPCa (red line) at various threshold probabilities are shown

The PSMA imaging-based risk score (AUC 0.933, 95% CI 0.878–0.988) showed excellent discrimination, outperforming the PRIMARY score (0.873) and the miPSMA score (0.838) in the test set (Fig. 3a). We note that the PSMA risk score incorporates more input variables (lesion diameter, SUVmax, SUVmax ratio, zone, and diffuse uptake) than the PRIMARY score (mainly visual, with grade 5 defined by SUVmax > 12) and the miPSMA score (which compares uptake with reference organs) and the comparison should be interpreted accordingly (Supplementary Table S5). Calibration analysis of the PSMA score in the test set showed good agreement between predicted and observed probabilities (Hosmer-Lemeshow *P* = 0.305, mean absolute error = 0.064; Fig. 3b).

We then developed a clinical model incorporating the PSMA score, PI-RADS, and PSAD (Supplementary Fig. S6) and explored its performance in the test set (*n* = 92). Predicted probabilities were categorized by the 20th and 80th percentiles of the training set into low (< 0.022, *n* = 18), intermediate (0.022–0.736, *n* = 53), and high (> 0.736, *n* = 21) risk categories. A simulated risk-guided strategy (Table 2; low-risk: no biopsy; intermediate-risk: SB + tri-modal fusion biopsy; high-risk: direct definitive treatment without prior biopsy (exploratory)) reduced the number of biopsies from 92 to 53 (42.4%), detected 43 csPCa cases (46.7%), missed one (2.3%), and resulted in no overdiagnoses. Decision curve analysis (Fig. 3c) and clinical impact curves (Fig. 3d) confirmed a positive net benefit across threshold probabilities of 5%-80%.

**Table 2:** Comparison of the current biopsy strategy and the proposed risk-guided biopsy strategies in the test set

Strategy	Biopsies	csPCa detected	csPCa missed	Overdiagnosis	Correctly avoided	Detection rate (%)	Miss rate (%)	Overdiagnosis rate (%)	Correct avoidance rate (%)
Current	92	40	4	0	0	43.5	9.1	0	0
New	53	43	1	0	39	46.7	2.3	0	42.4

Current strategy: SB + MRI-US fusion biopsy; New strategy: low-risk (no biopsy), intermediate-risk (SB + tri-modal fusion biopsy), high-risk (direct definitive treatment without prior biopsy (exploratory))

### Sensitivity analyses and safety

Sensitivity analyses (Supplementary Tables S6–S13, Supplementary Fig. S7) yielded results consistent with the primary findings. No serious adverse events occurred. Gross hematuria was observed in 34.0% of patients on day 1, declining to 11.2% on day 3 and 1.1% at 1 week. All cases were self-limiting. Post-procedural fever (≥ 38 °C) occurred in two patients (0.6%) on the day of biopsy (maximum temperatures 38.3 °C and 38.5 °C); both conditions resolved with oral levofloxacin and did not require hospitalization. One patient (0.3%) developed acute urinary retention requiring temporary catheterization, which was removed after 1 week. No episode of sepsis, rectal bleeding, or hematospermia requiring intervention was recorded. No patient required intravenous sedation or post-procedural analgesics.

## Discussion

In this prospective paired diagnostic accuracy study, tri-modal PSMA PET/CT-MRI-US fusion biopsy detected 4.9% more csPCas than MRI-US fusion biopsy (McNemar *P* < 0.001; NNB 20.5). The improvement was greatest for PSMA-positive lesions smaller than 10 mm (8.3%) and PI-RADS 4 lesions (5.8%), precisely the subgroups in which diagnostic uncertainty is greatest. Incorporating PSMA information into the fusion biopsy platform thus provides clinically meaningful incremental value beyond mpMRI alone.

It is important to place these findings in the context of the role of MRI. MRI remains indispensable within the tri-modal framework. It provides the anatomical reference for surgical planning, guides transperineal biopsy, and characterizes tumor aggressiveness through PI-RADS scoring and multiparametric sequences [17]. Our data showed that 68.5% (137/200) of PSMA-positive lesions had corresponding MRI-visible targets (PI-RADS ≥ 4), and 72.3% (149/206) of csPCa lesions were MRI-positive. The 137 MRI-only lesions included 24 csPCa cases (17.5%), confirming MRI’s independent diagnostic contribution. PSMA PET/CT does not replace MRI but provides complementary molecular information that identifies the 27.7% of csPCa lesions missed by MRI alone. A purely PSMA-US fusion approach would lack the anatomical detail and T2-weighted and diffusion-weighted imaging characterization provided by MRI, potentially increasing the rate of false-positive targeting. Future studies should directly compare PSMA-US fusion biopsy alone with tri-modal fusion biopsy to quantify this incremental benefit.

PSMA PET exploits receptor overexpression in aggressive prostate cancer to identify lesions that are occult on MRI [15, 18–20]. In our cohort, 39.3% of PSMA-only lesions were csPCa compared with 17.5% of MRI-only lesions, consistent with prior reports [7, 10, 16]. The GEE analysis demonstrated the independent predictive value of PSMA beyond MRI, with a fully adjusted OR of 1.97 (95% CI 1.27–3.08; *P* < 0.001). There was no significant modality-by-zone interaction (*P* = 0.511), indicating that transition zone signal heterogeneity due to benign prostatic hyperplasia did not impair fusion accuracy. By directly converting molecular uptake into biopsy coordinates through the fusion platform, the tri-modal approach avoids the uncertainty inherent in visual side-by-side image correlation.

The nonsignificant advantage of tri-modal fusion for PI-RADS 5 lesions likely reflects a ceiling effect. In this subgroup, MRI-US fusion biopsy had already achieved a high csPCa detection rate, leaving limited room for PSMA PET to provide additional diagnostic value. This finding is consistent with the PRIMARY trial [8]. Conversely, the greater improvement observed for PI-RADS 4 lesions (5.8%) reflects the subgroup with the greatest diagnostic uncertainty, in which PSMA PET most effectively reclassifies risk. This finding is supported by the cost-effectiveness analysis of Privé et al. [21].

The PSMA-derived risk score combined imaging biomarkers that capture distinct dimensions of tumor aggressiveness. Lesion size reflects tumor burden, SUVmax quantifies receptor expression, and lesion location accounts for anatomical differences in PSMA uptake. This multibiomarker approach may explain why the score (AUC 0.933) outperformed single-modality systems [22]. In a retrospective simulation using the test set, a clinical model combining the PSMA score with PI-RADS and PSAD would have enabled biopsy deferral in 42.4% of patients while missing only 2.3% of csPCas, below the 5% threshold recommended by current guidelines [15]. We emphasize that this was an exploratory, retrospectively simulated analysis that requires prospective validation. The risk thresholds are cohort-specific and should not be applied clinically without external validation.

We used 18 F-PSMA-1007 rather than 68Ga-PSMA-11. The longer half-life and wider availability of 18 F-PSMA-1007 come at the cost of higher pelvic background activity due to hepatobiliary excretion [23]. The PRIMARY trial used 68Ga-PSMA-11 [8]; however, the uptake patterns for csPCa detection are comparable between the two tracers [23, 24]. The lesion-level findings reported here should therefore be generalizable across tracers, although direct head-to-head comparisons are warranted.

Adding PSMA PET/CT to the diagnostic pathway incurs additional costs and radiation exposure (approximately 3–5 mSv for ^18^F-PSMA-1007, comparable to 1 year of background radiation). In current clinical practice, not all patients with suspected prostate cancer routinely undergo PSMA PET/CT before biopsy. The modality is typically reserved for patients with a prior negative biopsy, equivocal MRI findings, or high-risk disease requiring staging. However, the risk-guided strategy offers a potential offset. In the test set, 42.4% of patients could have avoided biopsy entirely, thereby reducing the cumulative costs of the biopsy procedure, pathological evaluation, and management of biopsy-related complications. The 4.9% incremental increase in csPCa detection may reduce downstream costs associated with delayed diagnosis and repeat biopsies. The NNB of 20.5 suggests that approximately 20 patients would need to undergo an additional PSMA PET/CT scan and tri-modal fusion biopsy to detect one additional case of csPCa. Privé et al. [21] recently reported that PSMA PET/CT may be cost-effective for selected subgroups, particularly male patients with PI-RADS 3 lesions. A formal economic evaluation incorporating region-specific cost data, quality-adjusted life years, and long-term oncological outcomes is needed before definitive conclusions can be drawn.

This study has several limitations. First, the superset design meant that tri-modal fusion biopsy sampled all MRI-positive lesions plus additional PSMA-only lesions. Therefore, the 4.9% increase in csPCa detection reflects both PSMA-guided targeting and increased sampling (321 additional cores), rather than a direct comparison between imaging modalities. Although the GEE analysis (adjusted OR 1.97, *P* = 0.003) showed that PSMA positivity independently predicted csPCa at the lesion level, statistical adjustment cannot substitute for a study design that controls sampling density. A matched-core design, in which the number of biopsy cores per lesion is fixed across strategies, would address this limitation in future studies.

Second, we cannot distinguish the effects of the AI registration platform from those of PSMA PET/CT itself. Whether the observed benefit resulted from improved spatial registration, more accurate needle placement, or the additional molecular information remains unclear. A factorial design (conventional MRI-US vs. AI-assisted MRI-US vs. AI-assisted tri-modal fusion) would help clarify these contributions. Electromagnetic needle tracking is susceptible to fiducial placement error, prostate deformation, and operator-dependent probe positioning. Because needle guidance accuracy was not formally validated, this represents another limitation. The risk-guided strategy was evaluated retrospectively in the test set rather than prospectively. Consequently, the estimated 42.4% reduction in biopsies and the NNB of 20.5 should be considered exploratory, and the proposed risk thresholds are specific to this cohort.

Finally, this was a single-center study, and the test set included only 92 patients. Additionally, the AI platform has not been evaluated across different operators or institutions. PSMA PET/CT is not yet routinely performed before biopsy, and its additional radiation exposure (3–5 mSv), cost, and logistical requirements remain important barriers to implementation. Moreover, an NNB of 20.5 indicates that the per-patient benefit is not large. Accordingly, this approach may be better suited for selected patients, such as those with prior negative biopsies or PI-RADS 3 lesions, rather than for routine use in all patients [21]. Finally, long-term follow-up was lacking. Biopsy findings were validated against immediate pathological results rather than long-term oncological outcomes, such as biochemical recurrence, and the long-term clinical significance of the additional PSMA-detected lesions therefore remains uncertain.

## Conclusions

In men suspected to have prostate cancer, tri-modal fusion biopsy increased csPCa detection by 4.9% compared to MRI-US fusion, particularly for patients with PSAD > = 0.15 or PI-RADS 4 lesions. A PSMA-derived risk score (AUC 0.933) enabled effective stratification, and a simulated risk-guided approach in the test set would have spared approximately 42% of unnecessary biopsies while missing only 2.3% of csPCa. These findings require multicentre validation before clinical adoption.

## Supplementary information

Below is the link to the electronic supplementary material.

Supplementary Material 1

## Data Availability

Deidentified individual participant data will be made available upon reasonable request to the corresponding author (sdfyymnwkyhh@163.com), subject to institutional ethics approval. The study protocol and statistical analysis plan are available from the corresponding author. Data will be available beginning 6 months after publication. Access criteria: proposals will be reviewed by the institutional review board; a signed data access agreement will be required.

## Declarations

### Artificial Intelligence Statement

During the preparation of this manuscript, the authors used AI-assisted tool (DeepSeek) for language polishing and grammar checking. The authors reviewed and edited all AI-generated suggestions and take full responsibility for the content of this manuscript.

## References

[1] Sathianathen NJ, Omer A, Harriss E, et al. Negative Predictive Value of Multiparametric Magnetic Resonance Imaging in the Detection of Clinically Significant Prostate Cancer in the Prostate Imaging Reporting and Data System Era: A Systematic Review and Meta-analysis. Eur Urol. 2020;78(3):402–14. 10.1016/j.eururo.2020.05.042. 32444265

[2] Stonier T, Simson N, Shah T, et al. The Is mpMRI Enough or IMRIE Study: A Multicentre Evaluation of Prebiopsy Multiparametric Magnetic Resonance Imaging Compared with Biopsy. Eur Urol Focus. 2021;7(5):1027–34. 10.1016/j.euf.2020.09.012. 33046412

[3] Cereser L, Evangelista L, Giannarini G, et al. Prostate MRI and PSMA-PET in the Primary Diagnosis of Prostate Cancer. Diagnostics (Basel). 2023;13(16):2697. 10.3390/diagnostics13162697. PMC10453091 37627956

[4] Otti VC, Miller C, Powell RJ, et al. The Diagnostic Accuracy of Multiparametric Magnetic Resonance Imaging Before Biopsy in the Detection of Prostate Cancer. BJU Int. 2019;123(1):82–90. 10.1111/bju.14420. 29804315

[5] Zattoni F, Morlacco A, Soligo M, et al. Diagnosis of Clinically Significant Prostate Cancer After Negative Multiparametric Magnetic Resonance Imaging. Cent Eur J Urol. 2022;75(3):277–83. 10.5173/ceju-2022-0079. PMC9628724 36381166

[6] Caracciolo M, Castello A, Urso L, et al. The Role of [68Ga]PSMA PET/CT for Clinical Suspicion of Prostate Cancer in Patients with or without Previous Negative Biopsy: A Systematic Review. Cancers (Basel). 2022;14(20):5036. 10.3390/cancers14205036. PMC9600353 36291820

[7] Mazzone E, Cannoletta D, Quarta L, et al. A Comprehensive Systematic Review and Meta-analysis of the Role of Prostate-specific Membrane Antigen Positron Emission Tomography for Prostate Cancer Diagnosis and Primary Staging before Definitive Treatment. Eur Urol. 2025;87(6):654–71. 10.1016/j.eururo.2025.03.003. 40155242

[8] Emmett L, Buteau J, Papa N, et al. The Additive Diagnostic Value of Prostate-specific Membrane Antigen Positron Emission Tomography Computed Tomography to Multiparametric Magnetic Resonance Imaging Triage in the Diagnosis of Prostate Cancer (PRIMARY): A Prospective Multicentre Study. Eur Urol. 2021;80(6):682–9. 10.1016/j.eururo.2021.08.002. 34465492

[9] Woo S, Becker AS, Leithner D, et al. Discordance Between Prostate MRI and PSMA-PET/CT: The Next Big Challenge for Primary Prostate Tumor Assessment? Eur Radiol. 2025;35(7):4043–54. 10.1007/s00330-025-11358-x. 39853335

[10] Chow KM, Lee A, Peh D, et al. Combined Prostate-specific Membrane Antigen Positron Emission Tomography and Multiparametric Magnetic Resonance Imaging for the Diagnosis of Clinically Significant Prostate Cancer. Eur Urol Oncol. 2025;8(5):1393–405. 10.1016/j.euo.2025.04.017. 40506357

[11] Lipkova J, Chen RJ, Chen B, et al. Artificial Intelligence for Multimodal Data Integration in Oncology. Cancer Cell. 2022;40(10):1095–110. 10.1016/j.ccell.2022.09.012. PMC10655164 36220072

[12] Austin PC. Bootstrap Model Selection had Similar Performance for Selecting Authentic and Noise Variables Compared to Backward Variable Elimination: A Simulation Study. J Clin Epidemiol. 2008;61(10):1009. 10.1016/j.jclinepi.2007.11.014. 17.e1. 18539429

[13] Seifert R, Emmett L, Rowe SP, et al. Second Version of the Prostate Cancer Molecular Imaging Standardized Evaluation Framework Including Response Evaluation for Clinical Trials (PROMISE V2). Eur Urol. 2023;83(5):405–12. 10.1016/j.eururo.2023.02.002. 36935345

[14] Carbon (Shenzhen) Medical Devices Co., Ltd., The First Affiliated Hospital of Soochow University. A Prostate Interventional Guidance Method, System, Storage Medium, and Electronic Device Based on PSMA PET, MRI and TRUS Multimodal Fusion. China patent CN119257741B. 2025 March 21.

[15] Mottet N, van den Bergh RCN, Briers E, et al. EAU-EANM-ESTRO-ESUR-ISUP-SIOG Guidelines on Prostate Cancer. European Association of Urology; 2024. 10.1016/j.eursup.2024.04.012.

[16] Rouvière O, Puech P, Renard-Penna R, et al. Use of Prostate Systematic and Targeted Biopsy on the Basis of Multiparametric MRI in Biopsy-naive Patients (MRI-FIRST): A Prospective, Multicentre, Paired Diagnostic Study. Lancet Oncol. 2019;20(1):100–9. 10.1016/S1470-2045(18)30639-0. 30470502

[17] Turkbey B, Rosenkrantz AB, Haider MA, et al. Prostate Imaging Reporting and Data System Version 2.1: 2019 Update of Prostate Imaging Reporting and Data System Version 2. Eur Urol. 2019;76(2):190–9. 10.1016/j.eururo.2019.02.033 30898406

[18] Cytawa W, Kircher S, Kübler H, et al. Diverse PSMA Expression in Primary Prostate Cancer: Reason for Negative [68Ga]Ga-PSMA PET/CT Scans? Immunohistochemical Validation in 40 Surgical Specimens. Eur J Nucl Med Mol Imaging. 2022;49(11):3938–49. 10.1007/s00259-022-05852-0. 35556160

[19] Bakht MK, Yamada Y, Ku S-Y, et al. Landscape of Prostate-specific Membrane Antigen Heterogeneity and Regulation in AR-positive and AR-negative Metastatic Prostate Cancer. Nat Cancer. 2023;4(5):699–715. 10.1038/s43018-023-00539-6. PMC10867901 37038004

[20] Bakht MK, Beltran H. Biological Determinants of PSMA Expression, Regulation and Heterogeneity in Prostate Cancer. Nat Rev Urol. 2025;22(1):26–45. 10.1038/s41585-024-00892-0. PMC11841200 38977769

[21] Privé BM, Govers TM, Israël B, et al. A Cost-effectiveness Study of PSMA-PET/CT for the Detection of Clinically Significant Prostate Cancer. Eur J Nucl Med Mol Imaging. 2025;52(9):3159–69. 10.1007/s00259-025-07190-6. PMC12222398 40072531

[22] Emmett L, Papa N, Hope TA, et al. Beyond Prostate Imaging Reporting and Data System: Combining Magnetic Resonance Imaging Prostate Imaging Reporting and Data System and Prostate-Specific Membrane Antigen-Positron Emission Tomography/Computed Tomography PRIMARY Score in a Composite (P) Score for More Accurate Diagnosis of Clinically Significant Prostate Cancer. J Urol. 2024;212(2):299–309. 10.1097/JU.0000000000004010 38758680

[23] Pianou NK, Stavrou PZ, Vlontzou E, et al. More Advantages in Detecting Bone and Soft Tissue Metastases from Prostate Cancer Using 18F-PSMA PET/CT. Hell J Nucl Med. 2019;22(1):6–9. 10.4103/0972-3919.359298. 30843003

[24] Gower-Fry L, Bailey JJ, Wuest M, et al. Development and Clinical Potential of 18F-PSiMA for Prostate Cancer PET Imaging. RSC Med Chem. 2025;16(8):3633–44. 10.1039/D4MD00275C. PMC12127850 40462771

