# Supplementary material for: Tri-modal PSMA PET/CT-MRI-US fusion biopsy for clinically significant prostate cancer: a prospective paired diagnostic study
Source: Eur J Nucl Med Mol Imaging. 2026 Sep 15;53(13):8610–20. doi: 10.1007/s00259-026-08181-x (PMC13727806; doi:10.1007/s00259-026-08181-x)
Supplement: Supplementary file 1 [file 259_2026_8181_MOESM1_ESM.pdf]

Supplementary Materials

Sensitivity Analyses

We conducted sensitivity analyses to test whether the primary findings held across alternative definitions and analytic approaches.

Unadjusted, multivariable, and partially adjusted GEE results

Results are presented in Tables S6, S7, and S8, respectively.

GEE with different correlation structures

The odds ratios for PSMA compared to MRI, using both independence and AR-1 correlation structures (Table S9), remained stable. For the independence structure, the odds ratio was 1.84 (95% CI 1.25–2.70); for the AR-1 structure, 1.86 (95% CI 1.29–2.69).

Alternative definitions of PET positivity

When PET positivity was redefined with a PRIMARY score of ≥4 or a miPSMA score of ≥2, instead of the previous thresholds of PRIMARY ≥3 or miPSMA ≥2, the absolute difference in clinically significant prostate cancer (csPCa) detection between the combined strategy and MRI-US fusion rose to 31.5% (95% CI 25–38%; P<0.001). This finding aligns with the primary analysis (Table S10).

Alternative definition of csPCa

Using an alternative csPCa definition (ISUP grade group ≥1 with >40% tumour involvement), the absolute difference observed was 8.1% (95% CI 1.8 –14.4%; P = 0.022), consistent with the primary analysis (Table S10).

Learning effect

The absolute differences in enrollment between the first and second halves were 3.2% (95% CI –1.0% to 7.5%) and 3.9% (95% CI 0.3–7.5%), respectively. These figures suggest there was no significant learning effect (Table S10).

PSMA score model validation

LASSO regression identified five key variables: maximum diameter, SUVmax ratio >1, peripheral zone involvement, diffuse transition zone uptake, and maximum SUVmax. Through ten-fold cross-validation, as shown in Table S11, the model achieved a mean AUC of 0.909 (range 0.842–0.990). Figure S1 presents the PSMA score nomogram, while Table S5 provides detailed performance metrics for the test set.

Bootstrap - derived confidence intervals for AUC differences

The PSMA score AUC exceeded that of PRIMARY by 0.06 (95% CI 0.009–0.117; P = 0.030) and that of miPSMA by 0.095 (95% CI 0.033–0.164; P = 0.006) (Table S12).

Alternative definitions of the combined strategy

When the combined strategy was redefined using PRIMARY ≥3 or miPSMA ≥2 to determine PET positivity, the absolute difference compared to SB was 43.2% (95% CI 37.5–48.9%). By excluding patients with a PSMA score near the cutoff range of 40–45, the difference was reduced to 36.2% (95% CI 30.5–42%). Both definitions yielded differences above 30%, confirming that the combined strategy outperformed MRI–US fusion regardless of threshold choice (Table S13).

miPSMA background selection

Using the spleen as the reference background for the miPSMA score resulted in an AUC of 0.791 (95% CI 0.740–0.841), which is not significantly different from the liver-based AUC of 0.793 (95% CI 0.742–0.843; DeLong P = 0.37; Figure S7). The clinical model nomogram is illustrated in Figure S6.

Additional detailed results

Across all sensitivity analyses (Tables S6–S13), the absolute difference between tri-modal and MRI–US fusion remained positive (range 3.2%–43.2%), and the direction of the lesion-level OR consistently favored PSMA over MRI.

Supplementary Figure Legends

Figure S1. PSMA score nomogram. Points are allocated based on the lesion's maximum diameter (in mm), whether the SUVmax ratio exceeds 1, involvement of the peripheral zone, and diffuse uptake in the transition zone. These total points are then used to estimate the predicted probability of clinically significant prostate cancer (csPCa).

Figure S2. Forest plot of csPCa detection rates by lesion group. Point estimates with 95% confidence intervals are shown for MRI-only, PSMA-only, and co-localised lesions. The dashed vertical line denotes the overall csPCa detection rate (42.3%). PSMA-only vs. MRI-only: OR = 3.04, 95% CI 1.69–5.47, $P < 0.001$, Woolf method. Co-localised vs. PSMA-only: OR = 3.26, 95% CI 1.94–5.49, $P < 0.001$, Woolf method.

Figure S3. Receiver operating characteristic (ROC) curve of SUVmax ratio for predicting csPCa in all PSMA-positive lesions. AUC = 0.735, 95% CI 0.673–0.797, $P < 0.001$, Mann-Whitney U test. The diamond marker indicates the optimal threshold (SUVmax ratio = 0.66, Youden index) with sensitivity = 0.66 and specificity = 0.77. The dashed diagonal line represents performance expected by chance (AUC = 0.5).

Figure S4. Box plot with individual data points of SUVmax ratio by csPCa status in all PSMA-positive lesions. The horizontal line within each box represents the median; box boundaries represent the interquartile range (IQR); whiskers extend to 1.5 × IQR. csPCa: median 0.8 (IQR 0.6–1.4); Non-csPCa: median 0.50 (IQR 0.4–0.7). $P < 0.001$, Mann-Whitney U test.

Figure S5. Subgroup analysis of csPCa detection in PSMA-only lesions by size and location. Bars represent the number of csPCa-positive (orange) and csPCa-negative (blue) lesions. Subgroups: (A) < 10 mm: 24/72 (33.3%), (B) ≥ 10 mm: 18/35 (51.4%), (C) Transition zone: 19/52 (36.5%), (D) Peripheral zone: 23/55 (41.8%). P values from Fisher's exact test. Size: $P = 0.092$; Zone: $P = 0.692$.

Figure S6. Clinical model nomogram. Points are allocated based on the PSMA score risk category (1, 2, 3), PI-RADS (3, 4, 5), and PSAD (ng/mL^2). These cumulative points indicate the predicted probability of clinically significant prostate cancer (csPCa).

Figure S7. miPSMA score comparison: liver vs. spleen background. ROC curves of miPSMA scores using liver or spleen as reference background. AUC values with 95% CIs are shown. DeLong test $P = 0.37$.

Supplementary Table Legends

Table S1. Inclusion and exclusion criteria.

Table S2. Schedule of events.
Timeline of all study procedures: enrollment, mpMRI, PSMA PET/CT, biopsy, results consultation, and radical prostatectomy (if applicable). · indicates that the procedure was completed as scheduled.

Table S3. Detailed subgroup analysis of the absolute difference in csPCa detection between combined strategy and MRI-US.
Data are presented as absolute difference (diff), 95% confidence interval (lower, upper), P value, interaction P value, and absolute difference expressed as percentage (diff perc). Interaction P values indicate heterogeneity across subgroups.

Table S4. Lesion characteristics for concordant and non-colocalized lesions.
Concordant: lesions visible on both MRI and PSMA PET/CT; PSMA-only: lesions visible only on PSMA PET/CT; MRI-only: lesions visible only on MRI. Data include number of lesions, csPCa detected (n and %), median diameter (mm), median SUVmax, and percentage of lesions in the peripheral zone.

Table S5. Performance of the PSMA score in the test set.
Patients are categorised into low (score ·7.1), intermediate (7.1·47.7), and high (>47.7) risk groups according to tertiles derived from the training set.

Table S6. Unadjusted GEE results for lesion-level csPCa detection.
Generalized estimating equations with exchangeable correlation structure. OR: odds ratio (PSMA vs. MRI). SE: standard error.

Table S7. Multivariable GEE results (adjusted for PSAD, age, and PI-RADS).
GEE with exchangeable correlation structure. OR: odds ratio. SE: standard error.

Table S8. Partially adjusted GEE results (adjusted for PSAD and age).
GEE with exchangeable correlation structure. OR: odds ratio (PSMA vs. MRI). SE: standard error.

Table S9. GEE analysis with different correlation structures.
Comparison of independence and AR-1 correlation structures. OR: odds ratio (PSMA vs. MRI). Lower/upper: 95% confidence intervals.

Table S10. Sensitivity analysis for the primary endpoint using alternative definitions of PET positivity and csPCa, and learning effect analysis.
Alternative PET definition: PRIMARY ·4 or miPSMA ·2. Alternative csPCa definition: ISUP grade group ·1 with >40% tumour involvement. CI: confidence interval.

Table S11. PSMA score sensitivity analysis.
LASSO-selected variables, mean AUC from 10-fold cross-validation, and AUC range.

Table S12. Bootstrap-derived 95% confidence intervals for the AUC difference between PSMA score and PRIMARY score, and between PSMA score and miPSMA score.
AUC difference, 95% CI, and DeLong P value are shown.

Table S13. Sensitivity analysis for the combined strategy using alternative definitions and after excluding patients near the cutoff.
Alternative definition: PRIMARY ·3 or miPSMA ·2 for PET positivity. Excluding patients with PSMA score 40·45 (near cutoff). CI: confidence interval.

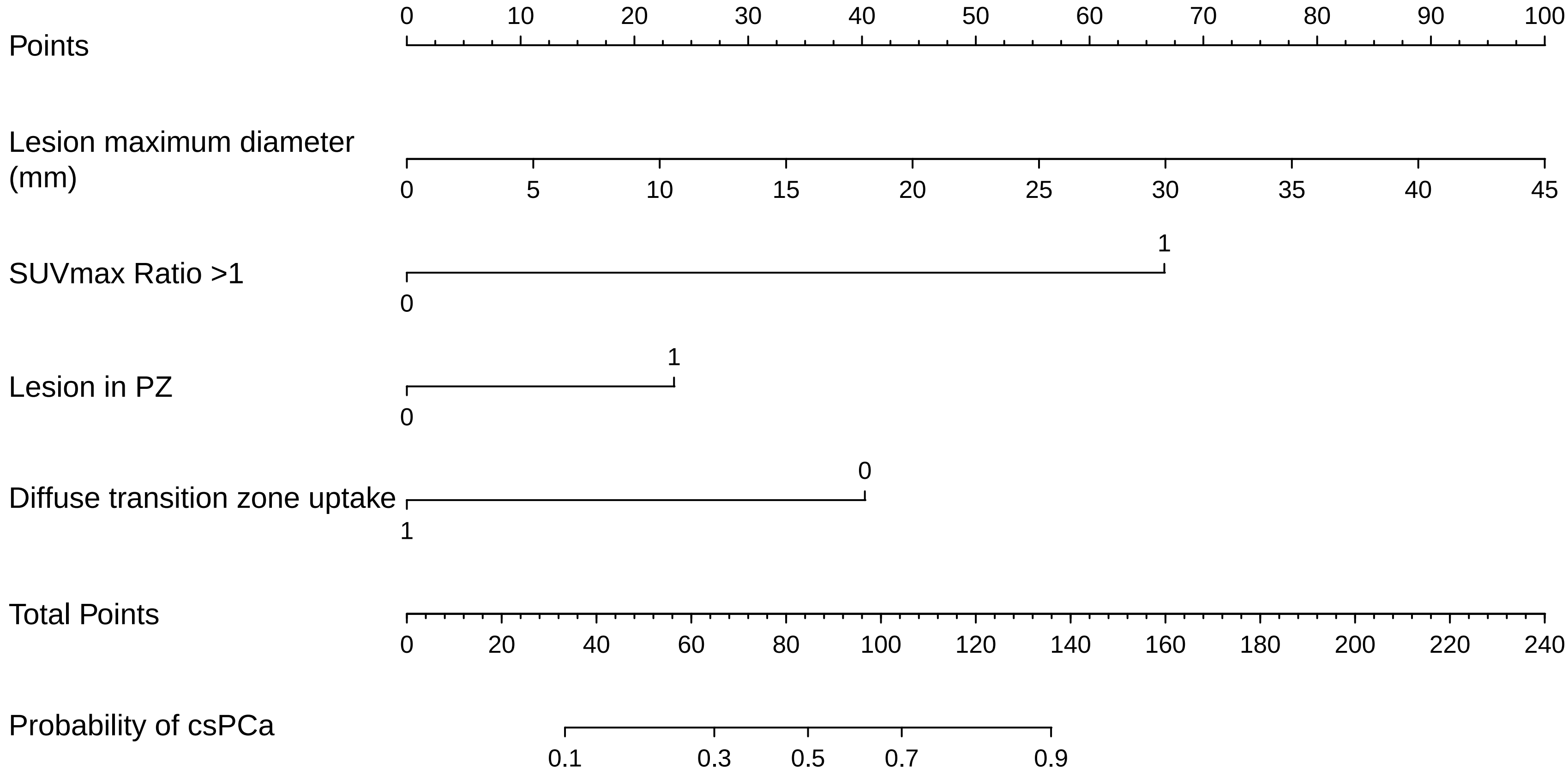

Figure S1. PSMA score nomogram

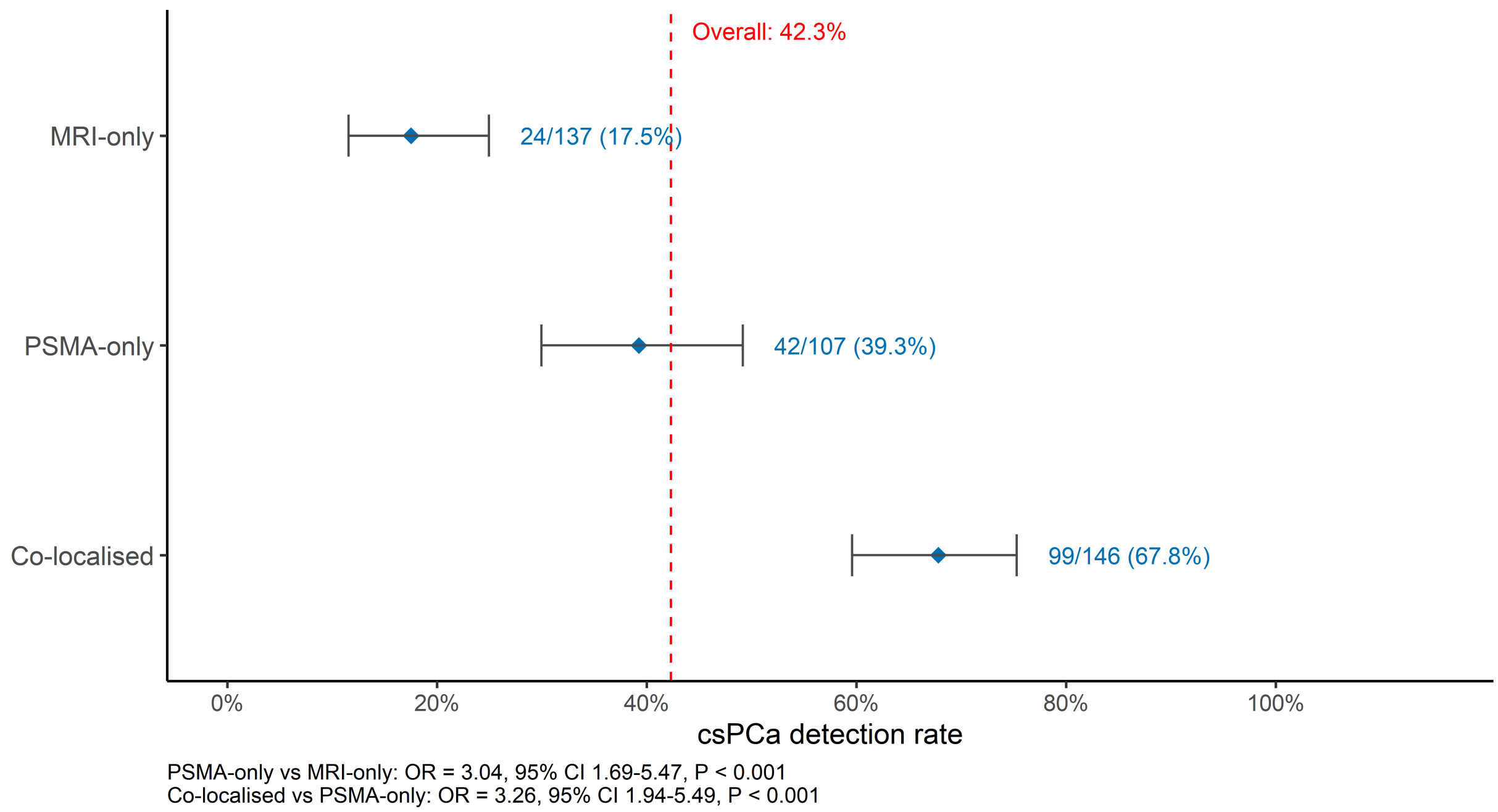

Figure S2. Forest plot of csPCa detection rates by lesion group

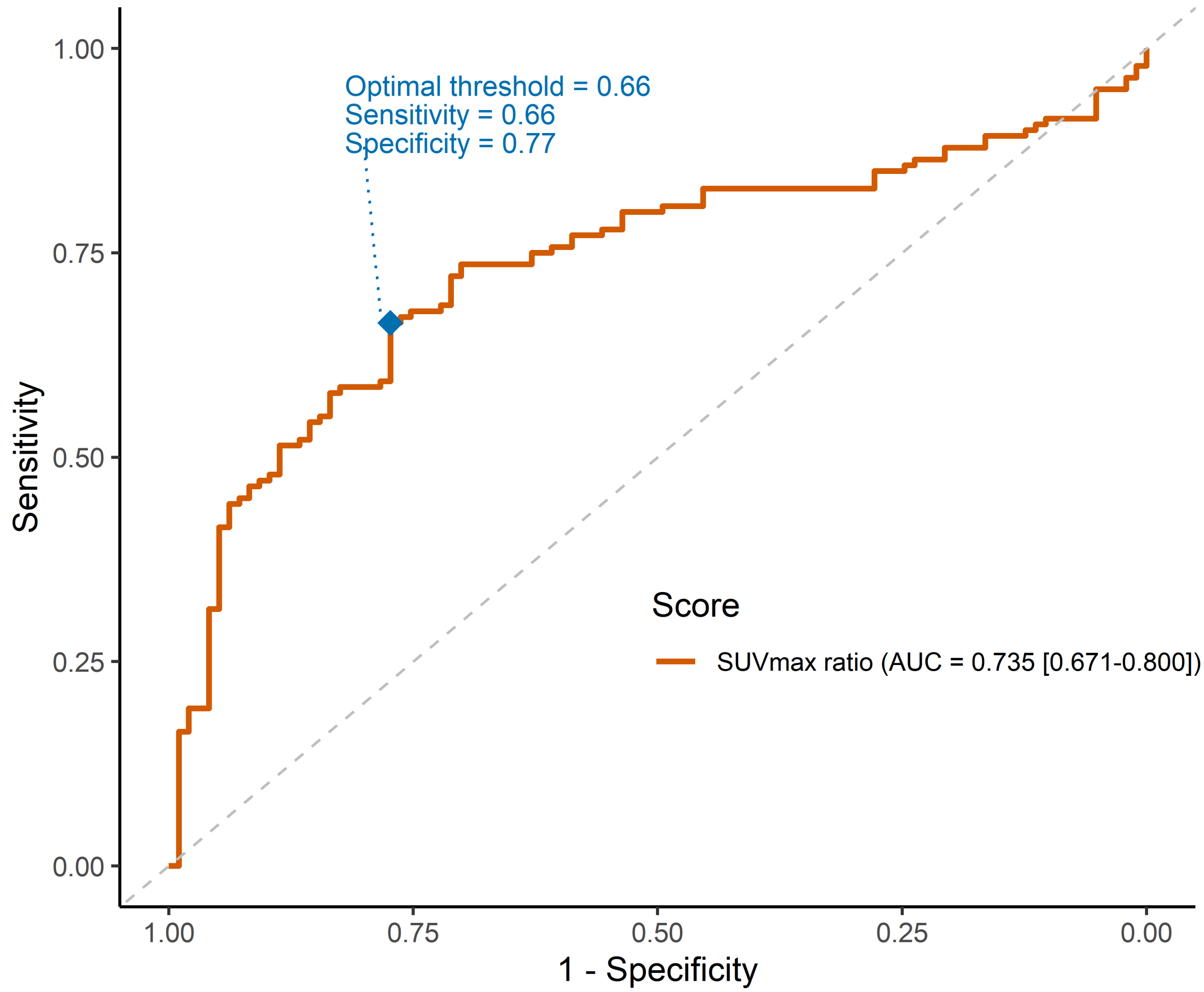

Figure S3. ROC curve of SUVmax ratio for predicting csPCa in all PSMA-positive lesions

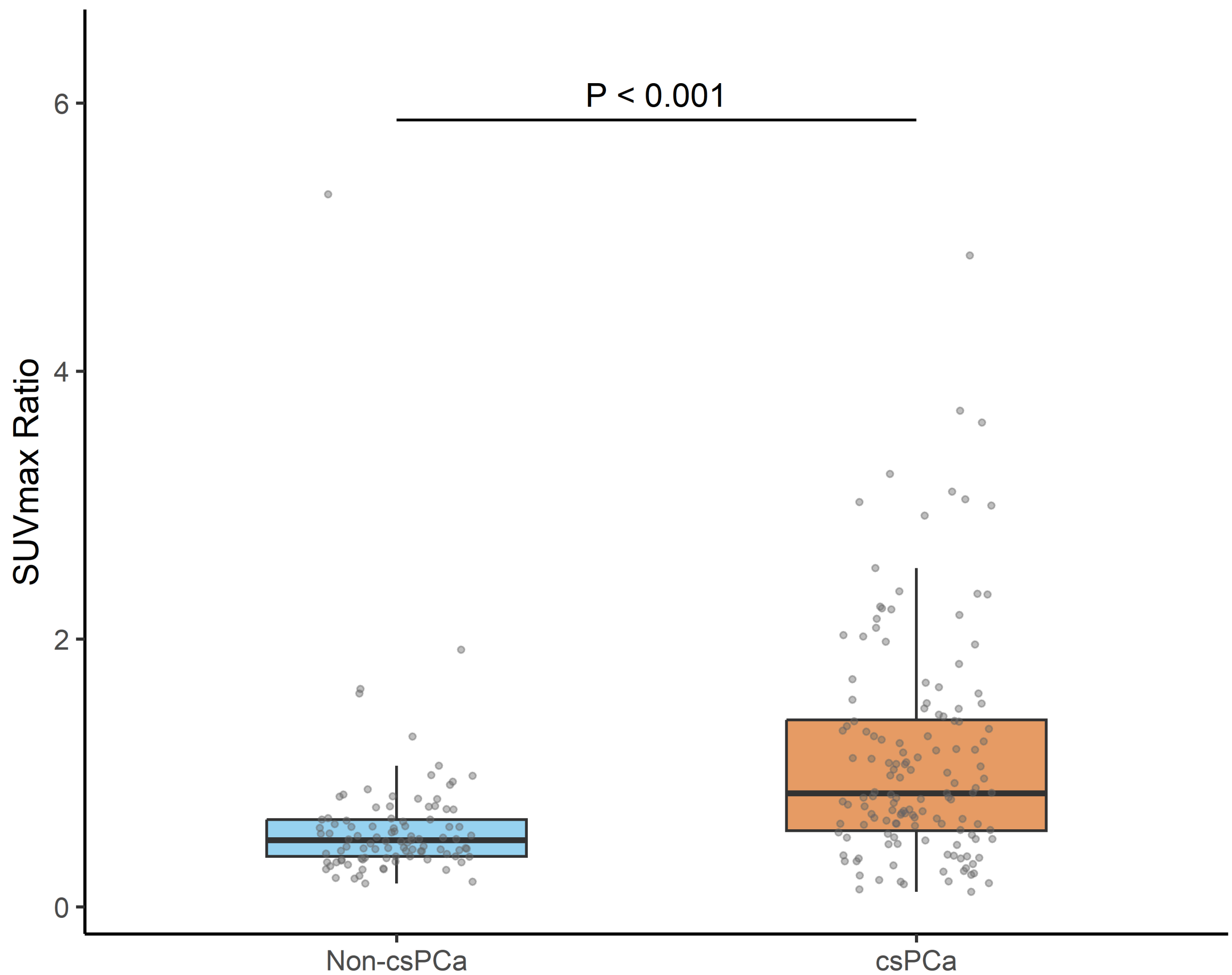

Figure S4. Box plot of SUVmax ratio by csPCa status in all PSMA-positive lesions

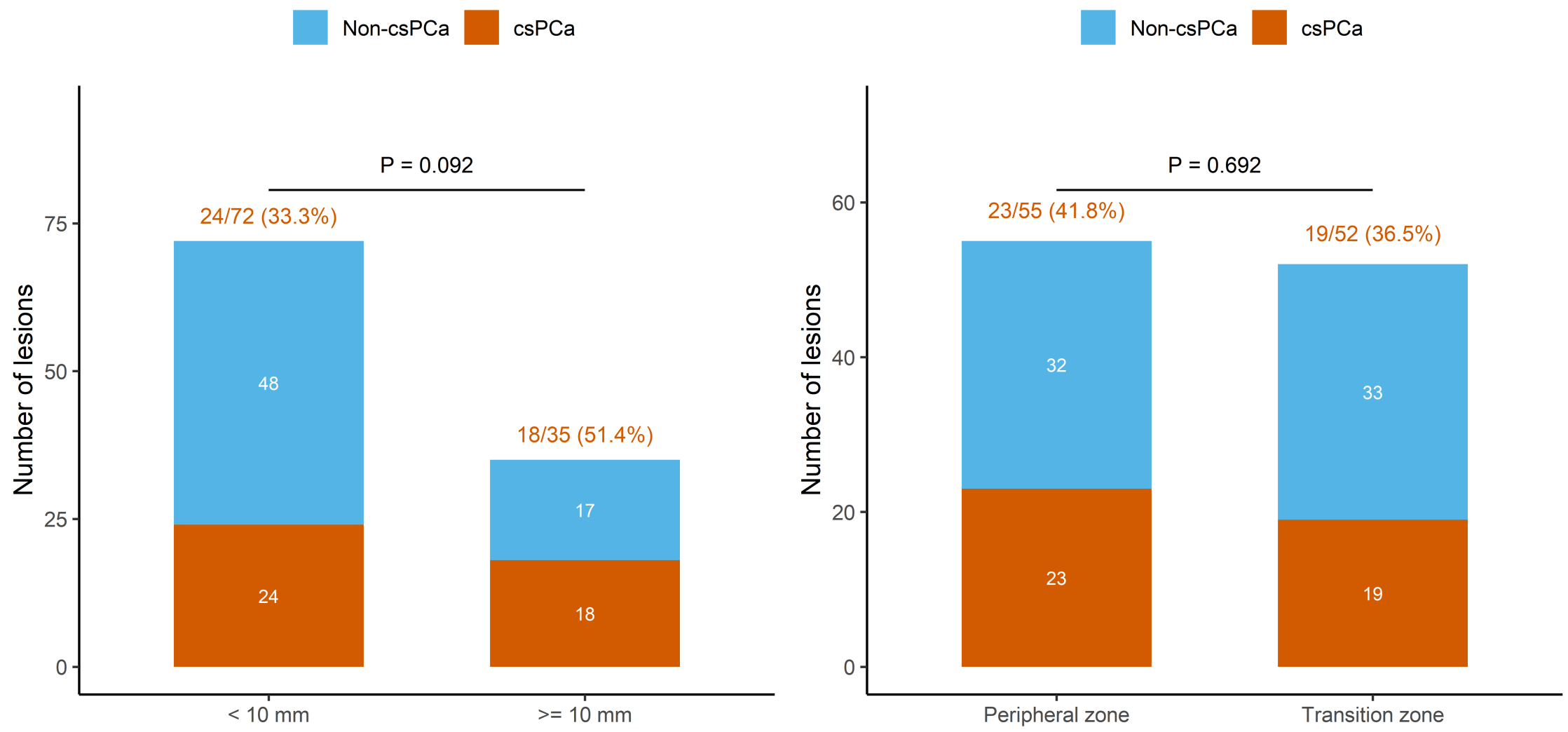

Figure S5. Subgroup analysis of csPCa in PSMA-only lesions

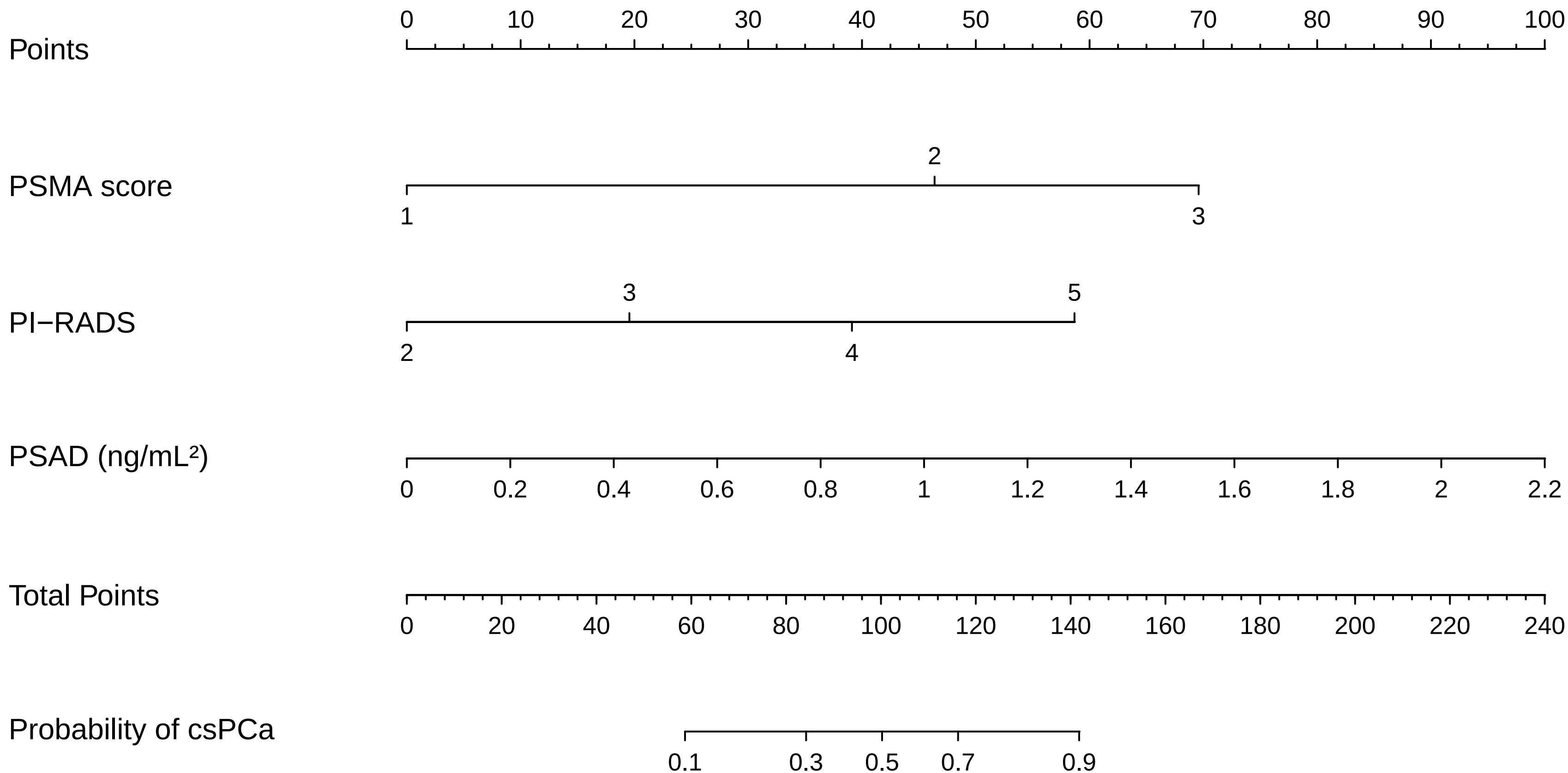

Figure S6. **Clinical model nomogram**

Figure S7. **miPSMA score comparison: liver vs. spleen background**

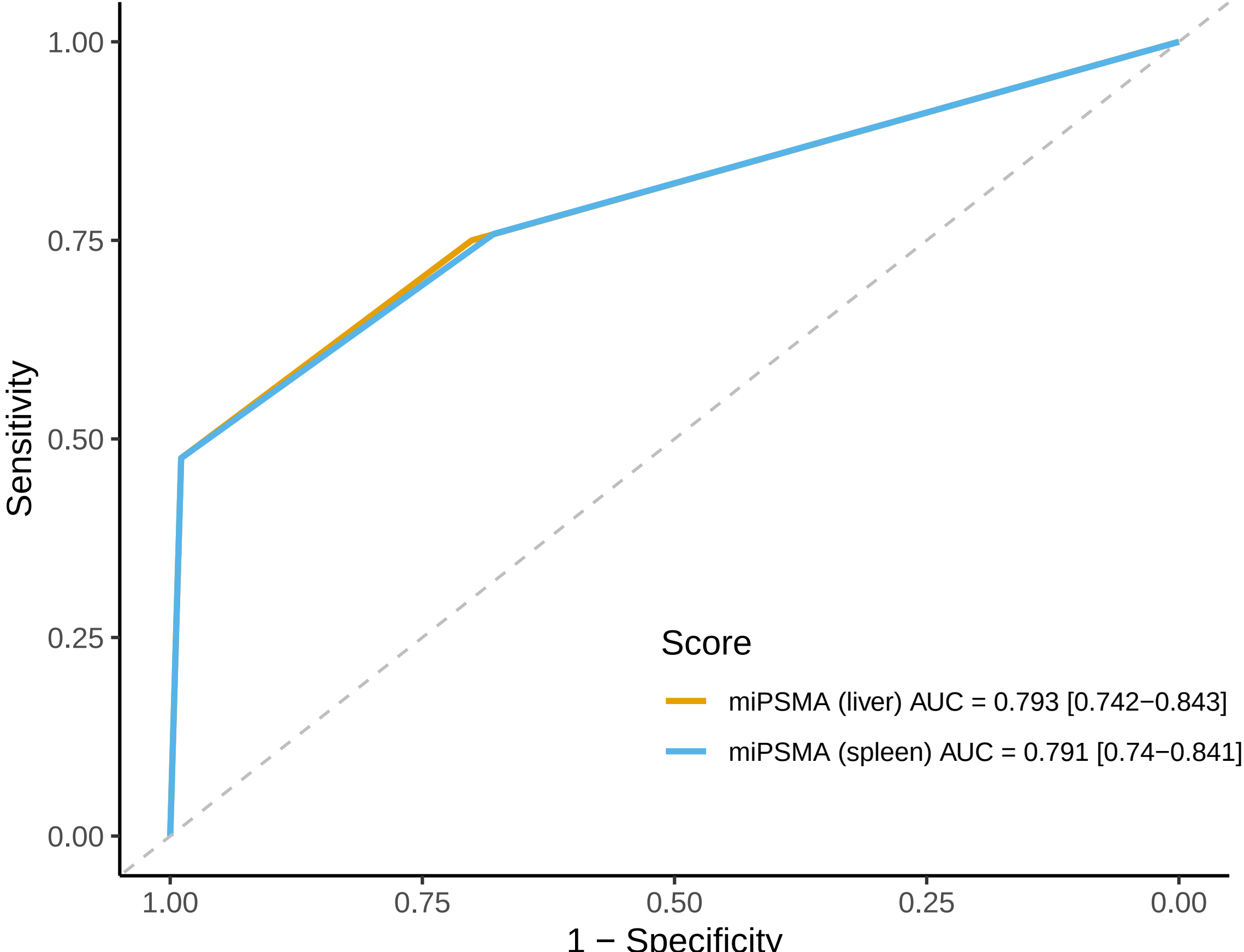

Table S1. Inclusion and exclusion criteria.

Inclusion criteria
1.Man who has a suspicion of prostate cancer with any of the following: a. Suspicious nodules in the prostate are found in DRE, any PSA value. b. Suspicious lesions are found in US or MRI, any PSA value. c. PSA>10 ng/ml, any f/tPSA and PSA density value. d. PSA4-10 ng/ml, abnormal f/tPSA value and PSAD value.
2.No previously diagnosed prostate cancer
3.No previously prostate biopsy
4.Scheduled for prostate biopsy
5.Can give written informed consent, participate and comply with the study
**exclusion criteria**
1.Previously diagnosed prostate cancer
2.Previously prostate biopsy
3.Inability/ incapacity to provide own consent
4.Contraindication to MRI, including but not restricted to:<br><br>Pacemaker or other electronic implant; Allergy or contraindication to MRI contrast, e.g. renal failure (eGFR <30mL/min), shrapnel, tattoos, non-removable body piercings (relative contraindication)
5.Researchers believe that patients who are not suitable to participate in this clinical trial

DRE: digital rectal examination; PSA: prostate specific antigen; US: ultrasound; MRI: magnetic resonance imaging; eGFR: Estimated Glomerular Filtration Rate

Table S2 Schedule of events.

List Interventions	Enrollment Visit	Visit MRI	Visit Nuclear medicine department	One-day admission for biopsy	Results consultation	Radical Prostatectomy
Inclusion/Exclusion criteria	√					
Informed Consent	√					
mpMRI		√				
PSMA PET/CT			√			
Prostate Biopsy (PSMA PET/CT-MRI-US fusion Targeted biopsy + systematic biopsy)				√		
Discuss subsequent intervention measures					√	
Analyze the histopathological data after radical surgery						If an assessment is necessary for radical prostatectomy and the patient's consent is obtained

√ indicates that the procedure was completed as scheduled.

Table S3 Detailed subgroup analysis of the absolute difference in csPCa detection between combined strategy and MRI-US.

Subgroup	diff	lower	upper	p	interaction p	diff perc	lower perc	upper perc
Overall	0.049	0.024	0.073	<0.001		4.870	2.406	7.335
PSAD ≥0.15	0.060	0.028	0.093	0.001	<0.001	6.047	2.760	9.333
PSAD <0.15	0.022	-0.008	0.051	0.480	<0.001	2.150	-0.830	5.131
PIRADS 3	0.045	0.006	0.085	0.074	<0.001	4.545	0.561	8.530
PIRADS 4	0.058	0.015	0.101	0.023	<0.001	5.785	1.499	10.071
PIRADS 5	0.028	-0.027	0.082	1	<0.001	2.778	-2.667	8.222
PSMA lesion <10mm	0.083	0.026	0.141	0.013	<0.001	8.333	2.559	14.108
PSMA lesion ≥10mm	0.064	0.017	0.112	0.023	<0.001	6.422	1.665	11.180
MRI lesion ≥10mm	0.036	0.009	0.062	0.023	<0.001	3.590	0.930	6.249
MRI lesion <10mm	0.083	0.017	0.150	0.041	<0.001	8.333	1.665	15.001
No MRI lesion	0.049	-0.019	0.116		<0.001	4.878	-1.882	11.639

Diff: absolute difference; lower/upper: 95% confidence interval bounds; diff perc: absolute difference expressed as percentage.

Table S4 Lesion characteristics for concordant lesions and non-colocalized PSMA-only and MRI-only lesions.

Lesion type	Lesions	csPCa detected	csPCa rate	Median diameter	Median SUVmax	Percentage of lesions in peripheral zone
Concordant	146	99	67.8	10	15.0	32.2%
PSMA-only	107	42	39.3	8	11.5	48.6%
MRI-only	137	24	17.5	12		50.4%

csPCa: clinically significant prostate cancer; SUVmax: maximum standardized uptake value.

Table S5 Performance of the PSMA score in the test set.

Risk level	N	csPCa detected	Detection rate (%)	Mean score	Score range	Max SUVmax	Lesions	Peripheral zone (%)	SUVmax ratio >1 (%)
1	29	1	3.4	2.5	≤7.1	0	0	0%	0%
2	23	8	34.8	31.5	7.1-47.7	9	1	34.8%	0%
3	40	35	87.5	81.7	>47.7	28.8	1.3	85%	62.5%

Mean score: mean PSMA score; Max SUVmax: maximum SUVmax of lesions; Lesions: average number of lesions per patient; Peripheral zone: percentage of lesions in peripheral zone; SUVmaxratio >1: percentage of lesions with SUVmaxratio >1.

Table S6 Unadjusted GEE results for lesion-level csPCa detection.

Variable	Coefficient	SE	Wald	P value	OR	OR lower	OR upper
(Intercept)	-0.413	0.126	10.78	<0.001	0.66	0.52	0.85
groupPSMA	0.622	0.188	10.95	<0.001	1.86	1.29	2.69

GEE: generalized estimating equations with exchangeable correlation structure. OR: odds ratio (PSMA vs MRI). SE: standard error.

Table S7 Multivariable GEE results (PSAD + age + PIRADS).

Variable	Coefficient	SE	Wald	P value	OR	OR lower	OR upper
(Intercept)	-8.277	1.296	40.76	<0.001	0	0	0
groupPSMA	0.68	0.226	9.04	0.0026	1.97	1.27	3.08
PSAD	3.405	0.691	24.29	<0.001	30.11	7.77	116.6
age	0.027	0.016	2.92	0.0873	1.03	1	1.06
PIRADS	1.326	0.185	51.41	<0.001	3.77	2.62	5.41

GEE: generalized estimating equations. OR: odds ratio. SE: standard error.

Table S8 Partially adjusted GEE results for lesion-level csPCa detection.

Variable	Coefficient	SE	Wald	P value	OR	OR lower	OR upper
(Intercept)	-4.23	1.062	15.86	<0.001	0.01	0	0.12
groupPSMA	0.623	0.208	8.99	0.003	1.86	1.24	2.8
PSAD	4.011	0.689	33.92	<0.001	55.22	14.32	213.01
age	0.037	0.015	6.31	0.012	1.04	1.01	1.07

GEE: generalized estimating equations. OR: odds ratio (PSMA vs MRI). SE: standard error.

Table S9 GEE analysis with different correlation structures.

correlation structures	OR	lower	upper	p
independence	1.839	1.254	2.697	0.002
ar1	1.863	1.289	2.692	<0.001

GEE: generalized estimating equations. OR: odds ratio (PSMA vs MRI).

Table S10 Sensitivity analysis for the primary endpoint using alternative definitions of PET positivity and csPCa, and learning effect analysis.

Analysis	Difference pct	Lower 95CI	Upper 95CI	P value
Alternative PET definition	31.5	25	38	<0.001
Alternative csPCa definition	8.1	1.8	14.4	0.022
Learning effect (early)	3.2	-1	7.5	NA
Learning effect (late)	3.9	0.3	7.5	NA

Alternative PET definition: PRIMARY ≥4 or miPSMA ≥2. Alternative csPCa definition: ISUP grade group ≥1 with >40% tumor involvement. Learning effect: comparison between first and second halves of enrollment (P values not applicable). CI: confidence interval.

Table S11 PSMA score sensitivity analysis. LASSO-selected variables, mean AUC from 10-fold cross-validation, and AUC range.

Analysis	Result
LASSO selected variables	max diameter,SUVmaxRatio > 1, lesion in PZ, TZ diffuse or no uptake, max SUVmax
Mean AUC (10 repetitions)	0.904
AUC range (10 repetitions)	0.866-0.934

max diameter: maximum lesion diameter (mm); any SUVmaxRatio gt1: presence of SUVmaxratio >1; any PZ: peripheral zone involvement; TZ diffuse or non uptake: diffuse uptake pattern; max SUVmax: maximum SUVmax of lesions; AUC: area under the curve.

Table S12 Bootstrap-derived 95% confidence intervals for the AUC difference between PSMA score and PRIMARY score, and between PSMA score and miPSMA score.

Comparison	AUC difference	Lower 95CI	Upper 95CI	Delong P
PSMA vs PRIMARY	0.06	0.009	0.117	0.030
PSMA vs miPSMA	0.095	0.033	0.164	0.006

AUC: area under the receiver operating characteristic curve; CI: confidence interval.

Table S13 Sensitivity analysis for the combined strategy using alternative definitions and after excluding patients near the cutoff.

Analysis	Difference pct	Lower 95CI	Upper 95CI
Alternative definition of combined strategy	43.2	37.5	48.9
Excluding patients near cutoff	36.2	30.5	42

Alternative definition: PRIMARY ≥3 or miPSMA ≥2 for PET positivity. Excluding patients with PSMA score 40–45 (near cutoff). CI: confidence interval.
